# Physiological View of the Nervous System, and Its Disorders

**Published:** 1868

**Authors:** E. A. Kunkler

**Affiliations:** Placerville, Cal.


### ARTICLE V.

*Physiological View of the Nervous System, and its Disorders.* By E. A. Kunkler, M. D., of Placerville, Cal.

Chorea.—This affection is characterized by irregular motions, or involuntary chronic contractions of some of the voluntary muscles; but some of the involuntary muscles are sometimes also involved, and even the intellectual faculties, in which case there is at the same time a certain degree of fatuity. Generally, however, there are only twitches of the muscles of the face, or convulsive contractions of the fingers, arms, or legs. But occasionally cases have been observed with violent contortions, rotary motions of the whole body, or an irresistible impulse to move in a determined direction, to leap, or to dance; whence this malady has also derived the name of St. Vitus' Dance, from a monastery in Germany, where some of such cases had formerly been cured.

In chorea volition is not suspended. The voluntary motions are regular, except those of the affected parts, which are interfered with by involuntary motions. These affect sometimes also the muscles of respiration, of deglutition, and of speech, producing more or less stammering.

The tremors in old age, in hard drinkers, in workers of mercury and lead, and in typhoid affections, which are generally considered to depend on general debility of the muscular and nervous systems, are probably but a derangement of the functions of the nerve-centre which co-ördinates or perfects the complicated voluntary motions, and are therefore a variety of chorea.

The experiments of M.M. Magendie, Flourens, and others, have indeed proved that when the cerebellum of animals is irritated, convulsive motions arise; that when it is injured, rotary motions—an irresistible impulse forward or backward is given to the animal, according to the particular place which has been hurt; and that when certain parts of the medulla oblongata are damaged, other kinds of involuntary motion are produced. The cerebellum, therefore, has been considered to be the nerve-centre by which the complicated muscular voluntary motions are co-ordinated, while the medulla oblongata, the spine, and the sympathetic ganglia, each in its own sphere, occasion the various involuntary motions. Hence there can be no doubt that, in the different forms of chorea, when voluntary muscles are disturbed in their motion, it proceeds from some lesion or irritation of some part of the cerebellum; that when the motion of the involuntary muscles is abnormal, either the medulla oblongata or the spine is involved; and that when the intellect is deranged, some part of the cerebrum proper must be disordered: these derangements causing an over-accumulation of arterial blood, or of oxygen, in the cells of the affected nerve-centre, and altering or preventing thereby the physiological action of these nerve-cells.

That in chorea, the mind and some of the voluntary and involuntary muscles are sometimes at the same time disordered, can be explained by the fact that many nerves are composed of one or more bundles of nerve-fibres, which, when afferent, are connected with nerve-cells possessed of different powers, seated in different nerve-centres; for the constiuent fibres of a nerve do not all run to the same point of termination. The irritation of such bundles of nerve-fibres, therefore, will be transmitted to different localities, and thereby create a variety of complex mental and motional phenomena.

This malady is sometimes combined with hysteria, or with epilepsy, when it assumes a mixed character. If uncomplicated, it does not in most cases manifest itself by regular, intermittent paroxyms, but generally its attacks last a certain time, without intermission.

Chorea is common among young people, from six to sixteen years of age—between the second teething and the age of puberty—but it may occur at any age. It is more often met with in girls than in boys. It is seldom severe and lasting, and scarcely ever proves fatal. In children it yields readily with the subsidence of the dental or intestinal irritation; and in girls, with the regular establishment of the catamenia, with which it is generally connected. However, any other source or cause of irritation may also produce this affection, especially injuries to the posterior part of the head, whereby the free action of the cerebellum is interfered with, or some part of its gray substance is altered.

A plethoric state alone is not likely to occasion chorea, but by supplying all the nerve-centres with an excess of arterial blood, it is apt to become a predisposing cause of this malady. In workers of mercury or of lead, the tremors are evidently the result of the absorption of these metals into the blood, by which they are carried to the nerve centres, especially to the cerebellum, when they become a source of irritation. In old age or in typhoid affections, they are clearly the effect of irritating substances, formed and retained in theb lood by imperfect elimination, whereby the nerve-centres receive an inadequate nutrition and are irritated at the same time.

In most persons who have died while they were affected with chorea, no lesion whatever has been found in the encephalon which could have been referred to this malady, though sometimes turgid bloodvessels, effusion in the ventricles, or some other alteration has been observed in the brain; probably in cases of long standing, by the long continuance of the irritation, these lesions or alterations had been produced.

For the empirical treatment of chorea, a variety of remedies have been recommended. However, it is obvious that such remedies can be beneficial only if they are capable of removing the irritating causes, or of correcting the organic derangements on which the nervous affections are dependent; and that in each case it is necessary, by a correct diagnosis, to determine the true pathological state, and to correct any organic derangement or other source of irritation by appropriate means.

*Case 1st.*—A boy, seven years of age, became affected by twitches of the muscles of his face, and his parents, not knowing what it was, consulted me on the subject. I found that his gums were swelled, and that he was cutting his molar teeth. This being clearly the cause of the nervous disturbance, I lanced his gums, and as soon as the dental irritation subsided, the twitches ceased also.

*Case 2d.*—A boy, five years of age, of a weak constitution, became affected by lateral twisting of his right leg, which interfered considerably with locomotion. Being informed by his mother that he was often rubbing his nose, and that during the night he was grinding his teeth, there could be no doubt of the existence of intestinal irritation, produced either by worms or by bad digestion. I therefore prescribed some Ol. Chenopodii, to be taken morning and night, to be followed the morning after by a brisk cathartic. Through its action no worms were brought away, but inasmuch as the nervous disturbance subsided, it was evident that it had depended on irritating matter in the gastro-intestinal canal, which was expelled by the purgative.

Sometimes chorea is connected with more complicated causes, which must be correctly considered, if we wish to be successful in its treatment.

*Case 3d.*—A young girl, fifteen years of age, of a delicate and weak constitution, became affected with twistings of her right arm, and her periods having not yet appeared, it was held by her physician that the protracted appearance of the menses and the nervous affection were both dependent on her general debility, for which different preparations of iron were prescribed. This treatment, however, failed to produce the desired effect.

When I saw this patient, I found—her complexion being yellowish and her lips red, not white—that there was no anemia. I ascertained also that her bowels were irregular and often constipated, that her spine was tender or sensitive on pressure at different places; and inasmuch as all the abdominal organs are directly or indirectly connected with the reflex action of the spinal nerves, it was evident that the sensitiveness of the medulla spinalis was the result of a protracted gastric, intestinal, and uterine irritation.

After the application of some cups to the spine, I prescribed gr. ii Hydrarg. c. creta, to be taken every night, to be followed in the morning by a Seidlitz powder, and ordered also some warm pediluvia. Thereby the skin reassumed soon its natural color, the bowels became regular, and the menses showed themselves, when the nervous disorder entirely vanished.

That amenorrhea is sometimes dependent on the presence of insufficient red corpuscles in the blood is admitted by everybody, and we have often seen such cases, in which the exhibitions of preparations of iron have proved entirely satisfactory. But the insufficiency of red globules in the blood, or anemia, is evinced by its peculiar physical signs, and when these signs of anemia are absent, and no such pathological condition exists, it is obvious that the administration of iron will prove not only negative, but sometimes even injurious. This metal has been, and is still, prescribed for weakness, almost on the same ground as the Chinese physician administers the powder of lion bone, namely, to impart to his debilitated patient some of the strength of the king of the forest. Without dwelling on such an unscientific conception, we will only remark that anemia is only one of the causes of weakness, when iron is required, but that weakness is also often the result of an oppressed circulation, by plethora, or by poison; of deleterious matter retained in the blood by imperfect elimination; and sometimes, also, of a strong shock to the nervous system, or of various other organic derangements, when, of course, iron cannot be of any benefit.

*Case 4th.*—A young lady, eighteen years of age, of a strong and full constitution, and clearly plethoric, who had been laboring for some time under dysmenorrhea, became affected with chronic contractions of her fingers, which, whenever she got hold of anything, would snap, and let the object slip. She had also a dull pain in the head, and by examining her spine, I found it not only very red, but also sensitive at different places on pressure. Otherwise she said that her bowels were regular, and her urinary functions normal.

That her dysmenorrhea, or functional disorder of the uterus, and her nervous affection, were both connected with her plethoric state, was beyond any doubt, as such a condition will cause not only engorgements in the nerve-centres, but also in the other organs; and withal, the excess of fibrin in the blood of plethoric persons will directly oppose the periodical flow which constitutes menstruation. But a plethoric state is always originated in imperfect disintegration and elimination, whereby the balance between assimilation and disintegration is disturbed, and inasmuch as such a disturbance is not possible when sufficient oxygen is admitted to the blood, and the functions of the lungs, liver, kidneys, and skin, are properly performed, it logically follows that in plethora there is, besides an excess of blood, also an abnormal state of this fluid, by insufficient or imperfect elimination; which, of necessity, will pervert the different secretions and excretions, and thus become a source of various irritations, especially of the organs seated in the splanchnic cavity, although the bowels and the urinary passages might appear to be in a normal condition.

Consequently, after the administration of a good cathartic, an ample venesection was performed, and some cups were applied along the spine. One Pil. hydrarg. was then prescribed, to be taken every night at bedtime, to be followed in the morning by a saline purgative. A solution of Pot. nit. with syrup was also ordered to be taken during the day, and plenty of active exercise in the open air recommended, in order to promote the activity of the lungs, and to keep the skin in a warm, soft, and moist condition. By this treatment the plethoric state gradually diminished, the uterine functions became normal, and the nervous disorder entirely disappeared.

Chorea may, as asserted by Dr. Andral, unquestionably be developed, or rather excited, by irritation, or by any strong mental impression; but in such cases there must be always some previous physical irritation which predisposes the nerve-centres to abnormal action, under the impulse, or by the vibration of any additional mental or physical shock. A plethoric state is, as we have already observed, a very common predisposing cause of this malady.

When chorea is connected with some incurable affection, or dependent on some lesion of the spine, or on some injury to the posterior part of the cranium, by which the free action of the cerebellum is hindered, or when some part of the cerebral matter is irritated, which cannot be remedied, a cure of the nervous affection cannot be expected. Such cases, however, though sometimes very inconvenient to the patient, are seldom of a serious character, and scarcely ever will prove fatal.

